# Three-Dimensional Speckle-Tracking Echocardiography-Derived Tricuspid Annular Properties in Acromegaly—Results from the MAGYAR-Path Study

**DOI:** 10.3390/biomedicines12071464

**Published:** 2024-07-02

**Authors:** Attila Nemes, Gergely Rácz, Árpád Kormányos, Nándor Gyenes, Nóra Ambrus, Csaba Lengyel, Zsuzsanna Valkusz

**Affiliations:** Department of Medicine, Albert Szent-Györgyi Medical School, University of Szeged, Semmelweis Street 8, P.O. Box 427, H-6725 Szeged, Hungary; racz.gergely@med.u-szeged.hu (G.R.); kormanyos.arpad@med.u-szeged.hu (Á.K.);

**Keywords:** echocardiography, tricuspid, annulus, three-dimensional, speckle-tracking, acromegaly

## Abstract

Introduction. Acromegaly is an endocrine pathology characterized by the overproduction of human growth hormone. The present study aimed to analyze three-dimensional speckle-tracking echocardiography (3DSTE)-derived tricuspid annular (TA) properties in detail in patients with acromegaly and to compare the findings to those of matched healthy controls. Methods. The present study consisted of 29 patients with acromegaly (mean age: 55.9 ± 14.5 years, 21 males), of which 13 had an active disease. The control population comprised 57 healthy subjects (mean age: 53.2 ± 8.4 years, 38 males). Results. In the presence of acromegaly, left atrial and end-diastolic left ventricular (LV) sizes were dilated, and LV ejection fraction was increased, which was accompanied by thickened interventricular septum and LV posterior wall as compared with matched healthy controls. The presence of grade 1 mitral (MR) and tricuspid (TR) regurgitations were more frequent in acromegaly than in controls, regardless of disease activity. Higher than grade 1 MR/TR was uncommon in acromegaly. The 3DSTE-derived all end-diastolic (2.47 ± 0.27 cm vs. 2.23 ± 0.27 cm; 8.73 ± 1.77 cm^2^ vs. 6.67 ± 1.40 cm^2^; 11.56 ± 1.34 cm vs. 10.20 ± 1.10 cm, *p* < 0.001 for all) and end-systolic (1.97 ± 0.27 cm vs. 1.77 ± 0.28 cm; 6.24 ± 1.61 cm^2^ vs. 5.01 ± 1.42 cm^2^; 9.80 ± 1.35 cm vs. 8.72 ± 1.10 cm, *p* < 0.001 for all) TA diameters, areas, and perimeters proved to be dilated, while TA functional parameters including TA fractional area change (28.77 ± 9.80% vs. 27.64 ± 15.34%, *p* = 0.720) and fractional shortening (20.60 ± 9.08% vs. 20.51 ± 8.81%, *p* = 0.822) were normal in acromegaly regardless of whether acromegaly was active or not. RA volumes respecting the cardiac cycle were dilated in acromegaly as compared with those of healthy controls regardless of disease activity and were associated with respective changes in TA dimensions. Conclusions. In the presented acromegaly patients, significant TA dilation with preserved function could be detected regardless of disease activity. RA volumes and TA dimensions are correlated in acromegaly.

## 1. Introduction

There is an increased scientific interest in cardiac mechanics and valvular abnormalities in specific disorders due to significant technical improvements in cardiac imaging, including echocardiography [1,2,3,4,5,6]. Three-dimensional (3D) speckle-tracking echocardiography (3DSTE) is capable of performing complex analysis of cardiac chambers and valvular alterations [3,4,5,6]. Acromegaly is an endocrine pathology characterized by the overproduction of human growth hormone (hGH) by a benign adenoma of the pituitary gland [7,8,9]. Nowadays, the focus is on the investigations of acromegaly-associated cardiovascular abnormalities [10,11,12,13,14,15,16,17,18,19]. Thanks to typical symptoms and a wide range of treatment options, currently, more advanced cases are rare [20]. However, potential significant volumetric and functional abnormalities of cardiac chambers have been described in recent studies, even in well-treated acromegaly patients. Left atrial (LA) volumes are increased in all phases of LA function with certain abnormalities in LA volume-based functional properties and LA strains, which can be accompanied by left ventricular (LV) hypertrophy, dilation, systolic, and diastolic dysfunction, impairment of strains and rotational mechanics. Moreover, mitral valve fibrosis, thickening, calcification, and regurgitation can be present together with dilation of the mitral annulus (MA) with preservation of its function. In the right heart, similarly to LA, the right atrium (RA) can be dilated in all phases of its function together with certain abnormalities in RA volume-based functional properties and strains. Right ventricular (RV) enlargement, hypertrophy, and dysfunction can also be acromegaly-related features. However, limited information was known about the tricuspid valve, including the fact that tricuspid regurgitation is not a typical feature of acromegaly and the ratio of different grades of tricuspid regurgitation is similar to that of controls [10,11,12,13,14,15,16,17,18,19]. The above-detailed alterations rightly raised the possibility that, similarly to MA abnormalities associated with left heart changes, alterations of the tricuspid annulus (TA) might also be associated with right heart abnormalities. Therefore, the present study aimed to perform a detailed analysis of 3DSTE-derived TA properties in cases with acromegaly and to compare their findings to those of matched healthy controls. The role of disease activity was also proposed to be examined.

## 2. Materials and Methods

**Figure 1:**
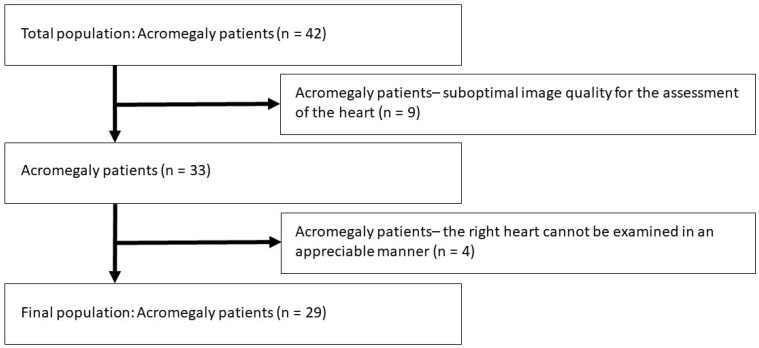
Flowchart of the study with the total acromegaly population and exclusions due to certain reasons.

**Patient population.** The present study identified a total of 42 acromegaly patients, from which 13 were excluded due to inferior image quality (Figure 1). The remaining group consisted of 29 patients with acromegaly being in sinus rhythm (mean age: 55.9 ± 14.5 years, 21 males), from which 13 had active disease. Diagnosis of acromegaly and its activity was judged by an expert endocrinologist based on current guidelines. The diagnosis of acromegaly was based on current clinical standards: typical clinical features, elevated hGH levels, and elevated insulin-like growth factor (IGF)-1 levels insuppressible with an oral glucose tolerance test. Acromegaly was considered active if serum hGH and/or IGF-1 concentration was over the diagnostic threshold [20,21,22]. All patients were cared for and treated by the local tertiary endocrine center, whose subdepartment is responsible for cases with severe/rare endocrine disorders, including acromegaly. None of the acromegaly patients were symptomatic from a cardiology point of view or had known cardiovascular disease. Sex-matched healthy volunteers were used as controls. The control population of 57 healthy subjects originates from a pool of healthy cases with a mean age of 53.2 ± 8.4 years (38 males). Subjects were considered healthy if they had no known disease and had no electrocardiographic and two-dimensional Doppler echocardiographic (2DE) abnormalities and if they did not take any medication regularly. In all patients and matched controls, complete 2DE and 3DSTE were completed at the same time. 3DSTE-acquired 3D echocardiographic datasets were stored and analyzed later offline. The presented work is part of the Motion Analysis of the Heart and Great Vessels bY Three-dimensionAl Speckle-tRacking Echocardiography in Pathological Cases (MAGYAR-Path) Study. This study was organized at our department to assess 3DSTE-derived TA properties among other parameters in certain pathologies (‘magyar’ means ‘Hungarian’ in the Hungarian language). The study was conducted in accordance with the Declaration of Helsinki (as revised in 2013) and approved by the Institutional and Regional Human Biomedical Research Committee of the University of Szeged, Hungary (No. 71/2011, prolonged 20 February 2023). All acromegaly patients and controls gave informed consent.

**Two-dimensional Doppler echocardiographic assessments.** During routine 2DE examinations, a commercially available Toshiba Artida^TM^ echocardiographic machine (Toshiba Medical Systems, Tokyo, Japan) was used attached with a 1–5 MHz PST-30BT phased-array transducer. All data analyses and quantifications followed recent guidelines. LV ejection fraction was calculated by using Simpson’s formula. Doppler assessments were used to exclude valvular regurgitations and stenosis and to determine early (E) and late (A) mitral inflow velocities [1,23].

**Three-dimensional speckle-tracking echocardiographic assessments.** The 3DSTE examination consisted of 2 steps. Firstly, 3D echocardiographic datasets were acquired by the same Toshiba Artida^TM^ echocardiographic tool (Toshiba Medical Systems, Tokyo, Japan) attached to a PST-25SX matrix-array transducer following image quality optimizations (gain, magnitude, etc.) [3,4,5,6]. Then, a full volume 3D ‘echocloud’ was acquired from the apical window focusing on the RA. As a second step, datasets were analyzed by a special, vendor-provided software package called 3D Wall Motion Tracking version 2.7 (Toshiba Medical Systems, Tokyo, Japan). The software automatically created 2 long-axis views [apical four-chamber (AP4CH) and two-chamber (AP2CH) longitudinal views] and 3 short-axis views at different levels of the RA [3,4,5,6].

**Figure 2:**
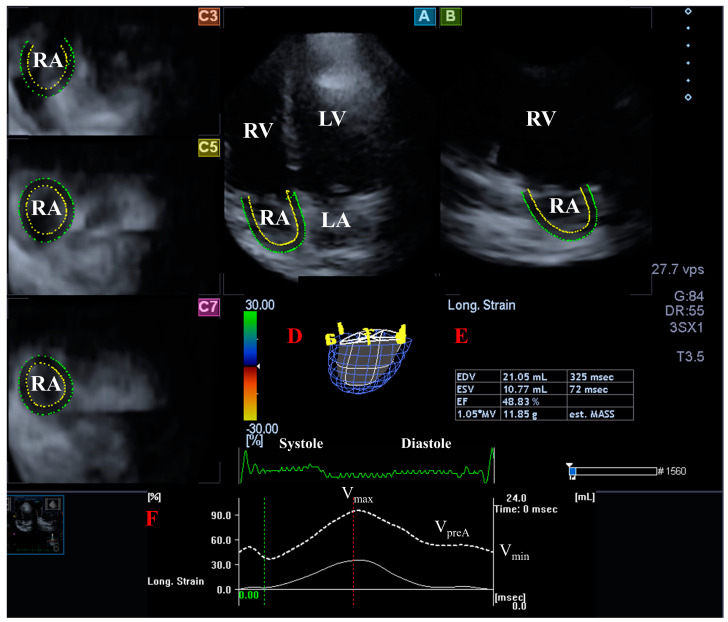
Assessment of the right atrium (RA) by three-dimensional (3D) speckle-tracking echocardiography: apical longitudinal four-chamber (A) and two-chamber views (B), and 3 short-axis views at basal (C3), midatrial (C5), and superior (C7) RA levels. Virtual 3D RA cast (D), RA volumetric data (E), time-global RA volume change curve (dashed white curve) and time-global RA longitudinal strain curve (white curve) respecting the cardiac cycle are also shown (F). Abbreviations. LA = left atrium, LV = left ventricle, RA = right atrium, RV = right ventricle, EDV = end-diastolic volume, ESV = end-systolic volume, EF = ejection fraction, est. = estimated, MV = myocardial volume. V_max_ = maximum right atrial volume, V_preA_ = volume at the onset of atrial systole, V_min_ = minimum right atrial volume.

**Measurement of RA volumes.** Among AP2CH and AP4CH views, 3 short-axis views at different RA levels were selected at basal, midatrial and superior RA levels. In end-diastole, several reference RA endocardial points were selected on the AP2CH and AP4CH views around the RA from the lateral to the septal TA edges, then automatic reconstruction was started for a sequential analysis when the complete endocardial RA surface was determined, and a virtual 3D cast of the RA was created (Figure 2). The following RA volumes were calculated: (1) maximum RA volume measured at end-systole, just before tricuspid valve opening (V_max_), (2) RA volume before atrial contraction determined at early-diastole at the time of P wave on the electrocardiography (V_preA_), and (3) minimum RA volume assessed at end-diastole, just before tricuspid valve closure (V_min_) [24].

**Figure 3:**
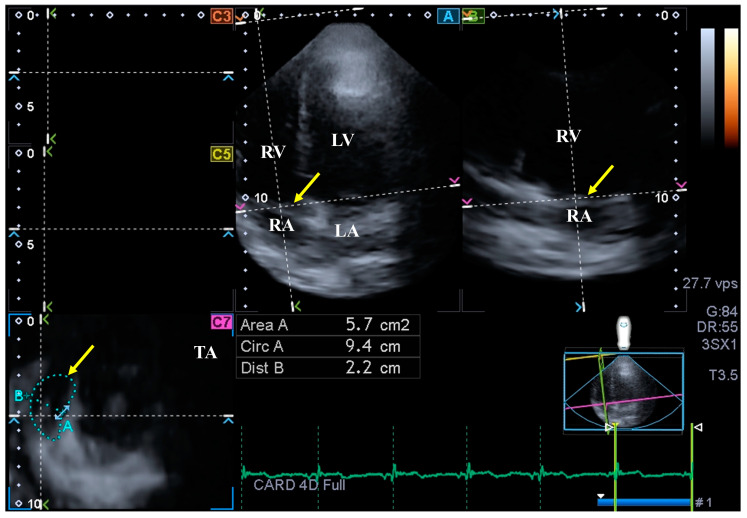
Extract from a three-dimensional full-volume dataset demonstrating the tricuspid annulus (TA) in an acromegaly patient: apical four-chamber (A) and two-chamber (B) views and a cross-sectional view at the TA level optimized in A and B views (C7). The yellow arrows represent the plane of TA. Abbreviations: LA = left atrium, LV = left ventricle, TA = tricuspid annulus, RA = right atrium, RV = right ventricle, Area = TA area, Circ = TA perimeter, Dist = TA diameter.

**Measurement of TA parameters.** When lateral and septal TA endpoints in AP2CH and AP4CH views were optimized, TA dimensions could be determined as an ‘en-face view’ on the C7 short-axis image (Figure 3) [25]. Several morphologic and functional TA properties were measured in end-systole and end-diastole:

- TA diameter (TAD) was defined as the perpendicular line drawn from the peak of TA curvature to the opposite side of the TA border;
- TA area (TAA) was assessed by planimetry;
- TA perimeter (TAP) was evaluated by planimetry;
- TA fractional shortening (TAFS) = [end-diastolic TAD − end-systolic TAD]/end-diastolic TAD × 100;
- TA fractional area change (TAFAC) = [end-diastolic TAA − end-systolic TAA]/end-diastolic TAA × 100.

**Statistical analysis.** Data were presented in different formats: mean ± standard deviation for continuous variables and number/percentage for dichotomous variables. *p* less than 0.05 was considered to be statistically significant. Fisher’s exact test was used for categorical variables. Student’s *t*-test with Welch correction and one-way analysis of variance (ANOVA) test with Bonferroni correction were used for continuous variables, where appropriate. Correlations were established by calculation of Pearson’s correlation coefficients. Bland–Altman method was used for assessing intra- and inter-observer agreements and intraclass correlation coefficients (ICCs) were also calculated. Statistical analyses were performed by SPSS software package software version 22 (SPSS Inc., Chicago, IL, USA).

## 3. Results

**Clinical data.** Demographic and laboratory findings and treatment used in acromegaly patients and controls are demonstrated in Table 1, together with differences between active and inactive acromegaly patients.

**Two-dimensional Doppler echocardiography**. In the presence of acromegaly, LA and end-diastolic LV dimensions were dilated, and LV-EF was increased, which was accompanied by thickened interventricular septum and LV posterior wall as compared with matched healthy controls. The presence of grade 1 mitral (MR) and tricuspid (TR) regurgitations were more frequent in acromegaly than in controls, regardless of disease activity. Higher than grade 1 MR/TR was infrequent in acromegaly (Table 2).

**Three-dimensional speckle-tracking echocardiography.** All end-diastolic and end-systolic TA dimensions were found to be dilated, while TA functional parameters were normal in acromegaly regardless of whether acromegaly was active or not. RA volumes respecting the cardiac cycle were dilated in acromegaly as compared with those of healthy controls regardless of disease activity (Table 3).

Acromegaly patients with hypertension had more dilated TA dimensions with preserved function and markedly larger RA volumes as compared with those of cases without hypertension. However, only end-diastolic TA area differed significantly between hypertensive patients with vs. without hypertension (Table 4).

**Correlations.** RA-V_max_ showed correlations with end-diastolic TAD (r = 0.56, *p* < 0.05), TAA (r = 0.62, *p* < 0.05), and TAP (r = 0.45, *p* < 0.05) and end-systolic TAD (r = 0.60, *p* < 0.05), TAA (r = 0.56, *p* < 0.05), and TAP (r = 0.40, *p* < 0.05). Similarly, RA-V_preA_ correlated with end-diastolic TAD (r = 0.45, *p* < 0.05), TAA (r = 0.60, *p* < 0.05), and TAP (r = 0.40, *p* < 0.05) and end-systolic TAD (r = 0.54, *p* < 0.05), TAA (r = 0.49, *p* < 0.05), and TAP (r = 0.42, *p* < 0.05). RA-V_min_ correlated with end-diastolic TAD (r = 0.71, *p* < 0.05), TAA (r = 0.73, *p* < 0.05), and TAP (r = 0.59, *p* < 0.05) and end-systolic TAD (r = 0.77, *p* < 0.05), TAA (r = 0.59, *p* < 0.05), and TAP (r = 0.61, *p* < 0.05), as well. hGH showed no correlations with any of the TA dimensions and functional parameters or with RA volumes. IGF-1 correlated with TAFS, with a correlation coefficient of 0.416 (*p* = 0.025). Otherwise, no correlation was found with other TA dimensions or RA volumes.

**Inter- and intra-observer variability of 3DSTE-derived parameters.** End-diastolic and end-systolic TA diameters, areas, and perimeters and end-systolic, early, and late diastolic RA volumes were calculated by 3DSTE, of which intra-observer agreement was assessed by measuring them twice by the same observer and inter-observer agreement was assessed by measuring them by two independent observers. Parameters were presented in mean ± SD format with ICCs, and the results are demonstrated in Table 5.

## 4. Discussion

Acromegaly is a rare chronic endocrine disorder that develops when the pituitary gland produces excess hGH and consequential IGF-1 during adulthood, leading to multisystem disease associated with increased morbidity and mortality [7,8,9]. In acromegaly, first hyperkinetic syndrome could be detected, then LV hypertrophy and diastolic dysfunction develop, leading to systolic functional deterioration and severe heart failure. Known symptoms are hypertension, various arrhythmias, coronary atherosclerosis, and valvular abnormalities as well [7,8,9,26,27,28]. In the left heart, reduced LV rotational mechanics with compensating LV contractility, complex left atrial volumetric and functional alterations, and dilated mitral annulus with its preserved function could be detected. In the right heart, the right atrium showed abnormalities as well as impaired right ventricular function [8,9,10,11,12,13,14,15,16,17,18,19]. However, tricuspid annular morphology and function have never been assessed by 3DSTE.

The TV or right atrioventricular valve incorporates fibrous, saddle-shaped TA, three leaflets, tendineal chords, and papillary muscles [29,30]. Imaging of TV is challenging, and 3DSTE was found to be capable of ‘en-face’ assessment of TA size and functional properties [2,3,4,5,6,25]. While TA seems to have a role in functional tricuspid regurgitation (FTR) [31], RA volume determines TA area in FTR as assessed by 3D echocardiography [32].

Clinically, disease-related TA abnormalities are worth looking at from three main aspects. First, the similarities and differences with the other atrioventricular valve, the mitral valve and its annulus (MA) should be considered. In a recent study, dilated MA could be demonstrated without its functional impairment in acromegaly patients and without significant functional mitral regurgitation (FMR). These abnormalities were present regardless of disease activity [13,14]. In the present study, TA showed similar dilation with preserved function, and these abnormalities were present regardless of disease activity. Although morphologic differences of atrioventricular valves and their annuli (for instance, in the number of leaflets, their fixing apparatus, etc.) are present with different shapes, sizes, and course of muscles of adjacent chamber areas (RA and RV), MA and TA showed similar abnormalities.

Second, the context of TA size and function with the cavities of the right heart, primarily RA, is an interesting clinical (patho)physiologic question. In recent studies, TA dilation was found to be associated with increased RA volumes in other pathologies and even in healthy subjects [32,33]. The present study confirmed that RA is dilated regardless of which phase of the cardiac function it was measured and was present regardless of disease activity. Moreover, strong correlations were found between TA parameters and RA volumes.

Third, the relationship between TA dilation and functional impairment and functional tricuspid regurgitation (FTR) should be an interesting topic for evaluation [32]. As mentioned before, abnormalities were already present in the case of MA in acromegaly patients without significant FMR [14]. In the present study, significant (larger than or equal to grade 2) FTR could not be detected in most of the acromegaly patients. Therefore, it could be stated that FTR cannot have any role in the determination of the above mentioned abnormalities. Additional effects of acromegaly-related tissue quality should be evaluated.

It could be stated that significant TA dilation with preserved function could be detected in acromegaly similar to MA, which is present regardless of disease activity. It could be theoretically explained by acromegaly-related hypertension and hormonal changes (IGF-1) and their effect on tissue quality and related to RA volumes. Compensatory effects of RA and RV functions could explain TA functional properties as well.

Over the past decade, imaging of the right heart has grown in importance. The presented findings could highlight more attention on the right heart abnormalities in acromegaly. The cardiac screening should focus more on the possible abnormalities of not only the RV but RA and related tricuspid valve, even in well-treated acromegaly. In the presence of any alterations, close monitoring may be considered. However, further studies are warranted in a larger patient population to confirm our findings with other imaging modalities.

### Limitation Section

- A relatively low number of patients with acromegaly were involved in the present study due to the rare nature of the disease. In Hungary with a population of almost 10 million inhabitants, appr. 300 patients with acromegaly are alive at the same time. The total population of acromegaly patients involved in the present study was 42, from which several subjects had to be excluded due to inferior image quality. The remaining group consisted of 29 acromegaly patients, as mentioned in the text. That means that more than 14% of the total Hungarian acromegaly population has been involved. All the patients were recruited from a tertiary endocrine center responsible for treatment, care and management of such endocrine disorders like acromegaly. At the time of examinations, the involvement of more patients was not possible due to the absence of more subjects.
- The 3DSTE-derived image quality is still lower than that of 2D echocardiography due to technical limitations like limited temporal and spatial resolution [3,4,5,6].
- During 3DSTE, no real 3D analysis was performed evaluating the saddle shape of TA. Only its 2D-projected evaluation was performed in selected 2D planes [25].
- The 3DSTE allows complex, detailed assessment of atria and ventricles. The present study did not aim to perform a volumetric and functional evaluation of such chambers [3,4,5,6].
- Although validation of 3DSTE-derived TA parameters was not purposed, inter- and intra-observer variability of data for TA parameters have been given.
- Age and classic cardiovascular risk factors, including hypertension, diabetes mellitus, and hypercholesterolemia, were more frequent in acromegaly patients as compared with controls, which could influence findings.
- Abnormalities in transmitral flow velocities could be partly explained by the presence of acromegaly-related hypertension.

## 5. Conclusions

In the presented acromegaly patients, significant TA dilation with preserved function could be detected regardless of disease activity.

## Figures and Tables

**Table 1:** Clinical and demographic data of acromegaly patients and controls.

	Controls	All Acromegaly Patients	*p* vs. Controls	Active Acromegalic Patients	*p* vs. Controls	Inactive Acromegalic Patients	*p* vs. Controls	*p* vs. Active Acromegaly
	(n = 57)	(n = 29)		(n = 13)		(n = 16)		
Clinical and demographic data								
Age (years)	53.2 ± 8.4	55.9 ± 14.5	0.0001	61.3 ± 11.1	0.03	51.4 ± 15.8	0.55	0.07
Male sex (%)	38 (67)	21 (72)	0.63	9 (69)	0.52	12 (75)	0.76	1.00
Hypertension (%)	0 (0)	18 (62)	0.0001	9 (69)	0.0001	9 (56)	0.0001	0.70
Diabetes mellitus (%)	0 (0)	6 (21)	0.001	4 (31)	0.0008	2 (13)	0.05	0.38
Hypercholesterolemia (%)	0 (0)	14 (48)	0.0001	7 (54)	0.0001	7 (44)	0.0001	0.71
Laboratory findings								
Serum hGH (ng/mL)	-	4.49 ± 5.83	-	5.12 ± 3.76	-	3.79 ± 7.01	-	0.54
Serum IGF-1 (ng/mL)	-	345.4 ± 301.5	-	417.9 ± 192.3	-	286.6 ± 363.1	-	0.25
Serum IGF-1 index	-	1.39 ± 1.01	-	1.84 ± 0.86	-	1.02 ± 0.99	-	0.03
Therapy								
Somatostatin analogue (%)	0 (0)	10 (34)	0.0001	6 (46)	0.0001	4 (25)	0.002	0.27
Bromocriptine (%)	0 (0)	10 (34)	0.0001	7 (54)	0.0001	3 (19)	0.009	0.06
Pegvisomant (%)	0 (0)	1 (3)	0.34	1 (8)	0.19	0 (0)	1.00	0.45
Hypophysectomy (%)	0 (0)	11 (38)	0.0001	7 (54)	0.0001	4 (25)	0.002	0.14

Abbreviations. hGH = human growth hormone, IGF-1 = insulin-like growth factor-1.

**Table 2:** Two-dimensional Doppler echocardiographic data of acromegaly patients and controls.

	Controls	All Acromegaly Patients	*p* vs. Controls	Active Acromegaly Patients	*p* vs. Controls	Inactive Acromegaly Patients	*p* vs. Controls	*p* vs. Active Acromegaly
	(n = 57)	(n = 29)		(n = 13)		(n = 16)		
LA and LV dimensions								
LA diameter (mm)	37.8 ± 4.9	42.7 ± 6.2	0.001	42.5 ± 7.5	0.009	42.8 ± 5.1	0.001	0.89
LV-EDD (mm)	48.5 ± 3.9	51.7 ± 5.4	0.003	51.0 ± 5.7	0.07	52.4 ± 5.3	0.003	0.50
LV-EDV (mL)	111.5 ± 33.5	132.2 ± 29.7	0.009	128.0 ± 29.0	0.11	135.9 ± 30.9	0.02	0.50
LV-ESD (mm)	32.5 ± 13.0	32.0 ± 4.9	0.70	30.9 ± 4.6	0.25	33.0 ± 4.6	0.69	0.26
LV-ESV (mL)	39.2 ± 13.0	43.2 ± 16.0	0.24	39.1 ± 13.6	0.94	46.7 ± 17.5	0.08	0.22
IVS (mm)	9.2 ± 1.4	10.3 ± 1.8	0.005	10.9 ± 2.0	0.001	9.8 ± 1.5	0.18	0.09
LV-PW (mm)	9.2 ± 1.6	10.9 ± 1.9	0.001	11.1 ± 2.0	0.001	10.8 ± 1.8	0.002	0.76
LV-EF (%)	64.6 ± 3.9	67.3 ± 7.3	0.04	68.8 ± 8.2	0.01	66.1 ± 6.5	0.28	0.33
E (cm/s)	71.5 ± 14.6	67.4 ± 15.3	0.25	58.3 ± 8.0	0.004	74.2 ± 16.1	0.55	0.004
A (cm/s)	64.7 ± 16.3	77.1 ± 15.9	0.002	76.0 ± 15.4	0.04	78.0 ± 16.6	0.007	0.75
Mitral regurgitation								
Mean MR grade	0.07 ± 0.26	0.58 ± 0.40	0.002	0.64 ± 0.41	0.001	0.25 ± 0.38	0.001	0.38
Grade 0 (%)	56 (98)	16 (55)	0.0001	8 (62)	0.0006	8 (50)	0.0001	0.71
Grade 1 (%)	1 (2)	11 (38)	0.0001	6 (46)	0.0001	5 (31)	0.002	0.47
Grades 2–4 (%)	0 (0)	2 (7)	0.11	2 (15)	0.03	0 (0)	1.00	0.19
Tricuspid regurgitation								
Mean TR grade	0.07 ± 0.26	0.58 ± 0.40	0.002	0.64 ± 0.41	0.001	0.25 ± 0.38	0.001	0.38
Grade 0 (%)	53 (93)	17 (59)	0.0002	6 (46)	0.0003	11 (69)	0.02	0.27
Grade 1 (%)	4 (7)	12 (41)	0.0002	7 (54)	0.0003	5 (31)	0.02	0.27
Grades 2–4 (%)	0 (0)	0 (0)	1.000	0 (0)	1.000	0 (0)	1.000	1.00

Abbreviations. LA = left atrium, LV = left ventricle, EDD = end-diastolic diameter, EDV = end-diastolic volume, ESD = end-systolic diameter, ESV = end-systolic volume, IVS = interventricular septum, PW = posterior wall, E and A = early and late diastolic mitral inflow velocities, MR = mitral regurgitation, TR = tricuspid regurgitation.

**Table 3:** Three-dimensional speckle-tracking echocardiographic data of acromegaly patients and controls.

	Controls	All Acromegaly Patients	*p* vs. Controls	Active Acromegaly Patients	*p* vs. Controls	Inactive Acromegaly Patients	*p* vs. Controls	*p* vs. Acromegaly
	(n = 57)	(n = 29)		(n = 13)		(n = 16)		
TA morphological parameters								
TAD-D (cm)	2.23 ± 0.27	2.47 ± 0.27	0.001	2.51 ± 0.21	0.001	2.44 ± 0.32	0.010	0.378
TAA-D (cm^2^)	6.67 ± 1.40	8.73 ± 1.77	0.001	9.06 ± 1.48	<0.001	8.47 ± 1.98	<0.001	0.867
TAP-D (cm)	10.20 ± 1.10	11.56 ± 1.34	0.001	11.51 ± 1.02	<0.001	11.59 ± 1.59	<0.001	0.456
TAD-S (cm)	1.77 ± 0.28	1.97 ± 0.27	0.001	1.93 ± 0.25	0.059	2.00 ± 0.29	0.005	0.907
TAA-S (cm^2^)	5.01 ± 1.42	6.24 ± 1.61	0.001	6.28 ± 1.49	0.005	6.21 ± 1.76	0.006	0.736
TAP-S (cm)	8.72 ± 1.10	9.80 ± 1.35	0.001	9.90 ± 1.07	0.001	9.72 ± 1.58	0.005	0.500
TA functional parameters								
TAFAC (%)	27.64 ± 15.34	28.77 ± 9.8	0.720	30.98 ± 10.12	0.459	26.98 ± 9.81	0.872	0.292
TAFS (%)	20.51 ± 8.81	20.60 ± 9.08	0.822	22.98 ± 10.22	0.380	17.69 ± 7.53	0.246	0.120
Right Atrial Volumes								
RA-V_max_ (mL)	45.37 ± 13.69	53.19 ± 13.95	0.044	51.06 ± 6.35	0.233	54.94 ± 18.17	0.063	0.550
RA-V_preA_ (mL)	36.03 ± 11.07	44.09 ± 11.01	0.010	44.01 ± 7.09	0.046	44.17 ± 13.78	0.047	0.975
RA-V_min_ (mL)	26.12 ± 8.27	34.20 ± 9.60	0.001	34.20 ± 7.68	0.011	34.20 ± 11.31	0.012	1.000

Abbreviations. TAD = tricuspid annular diameter, TAA = tricuspid annular area, TAP = tricuspid annular perimeter, D = end-diastolic, S = end-systolic, TAFAC = tricuspid annular fractional area change, TAFS = tricuspid annular fractional shortening, RA = right atrial, V_max_ = maximum systolic RA volume, V_preA_ = pre-atrial contraction early diastolic RA volume, V_min_ = minimum late diastolic RA volume.

**Table 4:** Three-dimensional speckle-tracking echocardiographic data of acromegaly patients with vs. without hypertension and controls.

	Controls	All Acromegaly Patients	*p* vs. Controls	Acromegaly Patients with Hypertension	*p* vs. Controls	Acromegaly Patients without Hypertension	*p* vs. Controls	*p* vs. Acromegaly Patients with Hypertension
	(n = 57)	(n = 29)		(n = 18)		(n = 11)		
TA morphological parameters								
TAD-D (cm)	2.23 ± 0.27	2.47 ± 0.27	0.001	2.53 ± 0.27	<0.001	2.38 ± 0.26	0.085	0.168
TAA-D (cm^2^)	6.67 ± 1.40	8.73 ± 1.77	0.001	9.26 ± 1.89	<0.001	7.87 ± 1.16	0.019	0.037
TAP-D (cm)	10.20 ± 1.10	11.56 ± 1.34	0.001	11.86 ± 1.51	<0.001	11.05 ± 0.84	0.019	0.111
TAD-S (cm)	1.77 ± 0.28	1.97 ± 0.27	0.001	1.98 ± 0.31	0.006	1.93 ± 0.18	0.062	0.618
TAA-S (cm^2^)	5.01 ± 1.42	6.24 ± 1.61	0.001	6.57 ± 1.69	<0.001	5.70 ± 1.39	0.144	0.159
TAP-S (cm)	8.72 ± 1.10	9.80 ± 1.35	0.001	10.08 ± 1.41	<0.001	9.35 ± 1.18	0.087	0.166
TA functional parameters								
TAFAC (%)	27.64 ± 15.34	28.77 ± 9.8	0.720	29.17 ± 9.74	0.694	28.13 ± 10.79	0.920	0.792
TAFS (%)	20.51 ± 8.81	20.60 ± 9.08	0.822	21.18 ± 9.56	0.788	18.24 ± 8.34	0.431	0.408
Right atrial volumes								
RA-V_max_ (mL)	45.37 ± 13.69	53.19 ± 13.95	0.044	54.13 ± 16.60	0.071	51.78 ± 9.59	0.215	0.723
RA-V_preA_ (mL)	36.03 ± 11.07	44.09 ± 11.01	0.010	45.51 ± 12.12	0.014	41.97 ± 9.47	0.165	0.496
RA-V_min_ (mL)	26.12 ± 8.27	34.20 ± 9.60	0.001	35.17 ± 10.41	0.003	32.76 ± 8.72	0.047	0.584

Abbreviations. TAD = tricuspid annular diameter, TAA = tricuspid annular area, TA*P* = tricuspid annular perimeter, D = end-diastolic, S = end-systolic, TAFAC = tricuspid annular fractional area change, TAFS = tricuspid annular fractional shortening, RA = right atrial, V_max_ = maximum systolic RA volume, V_preA_ = pre-atrial contraction early diastolic RA volume, V_min_ = minimum late diastolic RA volume.

**Table 5:** Intra- and inter-observer agreement for three-dimensional speckle-tracking echocardiography-derived tricuspid annular dimensions and right atrial volumes respecting the cardiac cycle.

	Intra-Observer Agreement		Inter-Observer Agreement	
	Mean ± 2 SD Difference in Values Obtained by 2 Measurements of the Same Observer	ICC between Measurements of the Same Observer	Mean ± 2 SD Difference in Values Obtained by 2 Observers	ICC between Independent Measurements of 2 Observers
End-diastolic TAD	0.01 ± 0.18 cm	0.95 (*p* < 0.0001)	0.03 ± 0.22 cm	0.95 (*p* < 0.0001)
End-diastolic TAA	−0.03 ± 1.11 cm^2^	0.95 (*p* < 0.0001)	0.03 ± 0.49 cm^2^	0.95 (*p* < 0.0001)
End-diastolic TAP	−0.02 ± 0.65 cm	0.96 (*p* < 0.0001)	−0.12 ± 0.62 cm	0.97 (*p* < 0.0001)
End-systolic TAD	−0.04 ± 0.29 cm	0.97 (*p* < 0.0001)	0.03 ± 0.42 cm	0.96 (*p* < 0.0001)
End-systolic TAA	−0.03 ± 0.27 cm^2^	0.96 (*p* < 0.0001)	−0.04 ± 0.59 cm^2^	0.97 (*p* < 0.0001)
End-systolic TAP	0.06 ± 0.49 cm	0.96 (*p* < 0.0001)	0.03 ± 0.51 cm	0.96 (*p* < 0.0001)
V_max_	1.1 ± 6.1 mL	0.97 (*p* < 0.0001)	1.2 ± 4.9 mL	0.94 (*p* < 0.0001)
V_preA_	−1.3 ± 7.9 mL	0.89 (*p* < 0.0001)	−1.4 ± 8.1 mL	0.89 (*p* < 0.0001)
V_min_	0.7 ± 5.4 mL	0.93 (*p* < 0.0001)	0.8 ± 4.1 mL	0.93 (*p* < 0.0001)

Abbreviations. ICC = interclass correlation coefficient, TAD = tricuspid annular diameter, TAA = tricuspid annular area, TAP = tricuspid annular perimeter, SD = standard deviation, V_max_ = maximum end-systolic right atrial volume, V_preA_ = pre-atrial contraction early diastolic RA volume, V_min_ = minimum end-diastolic right atrial volume.

## Data Availability

The manuscript has not been published elsewhere. Data are contained within the article.
